# Peptidomic Analysis of Potential Bioactive Peptides in Mare Milk Under Different Heat Treatment Conditions

**DOI:** 10.3390/foods13223592

**Published:** 2024-11-10

**Authors:** Xiaoxiao Lou, Wei Shao, Yating Wu, Hongpeng Ma, He Chen, Nan Zheng, Yankun Zhao

**Affiliations:** 1 Ministry of Agriculture and Rural Affairs-Laboratory of Quality and Safety Risk Assessment for Agro-Products, Agricultural Product Quality and Safety Risk Assessment Laboratory, Institute of Quality Standards & Testing Technology for Agro-Products, Xinjiang Academy of Agricultural Sciences, Urumqi 830091, China; louxiaoxiao1024@163.com (X.L.); yatingwu@xaas.ac.cn (Y.W.); mhp_0820@163.com (H.M.); hechen@xaas.ac.cn (H.C.); zhengnan_1980@126.com (N.Z.); 2 Xinjiang Meat and Milk Herbivore Nutrition Laboratory, College of Animal Science, Urumqi 830052, China; dksw@xjau.edu.cn; 3 Key Laboratory for Quality & Safety Control for Milk and Dairy Products of Ministry of Agriculture and Rural Affairs, Institute of Animal Science, Chinese Academy of Agricultural Science, Beijing 100193, China

**Keywords:** mare milk, bioactive peptides, temperature stage, biological activity

## Abstract

Active peptides in mare milk have unique biological activities, but how the bioactive protein in mare’s milk changes under the influence of temperature has not been fully studied. Therefore, in this study, the differential expression of bioactive peptides potentially present in horse milk under different heat treatment conditions was investigated for the first time using peptidomic and bioinformatic techniques. We collected a total of 15 samples. In this study, we divided the samples into five groups, specifically, 65 °C, 30 min; 72 °C, 15 min; 83 °C; 10 min; 95 °C, 5 min; and an untreated group as a control, which involved four different temperature treatments, in order to understand changes in bioactive peptides and to identify the sequence of bioactive peptides. In the experiment, a total of 2341 active peptides were identified. The amino acid composition of the potential active peptides remained stable across different temperatures, but their abundance varied with temperature. In all, 23 peptides from 20 different proteins were identified, with the highest number of active peptides identified at 72 °C. Through database searches, we found that a majority of these peptides were within β-lactoglobulin and immunoglobulin domain proteins, which are known for their potential biological activities. These findings provide a theoretical foundation for the development of peptides with different bioactivities as potential functional foods.

## 1. Introduction

Fresh mare milk is a distinctive grassland resource, characterized by its unique national features and a profound cultural background. It boasts a higher nutritional value than other dairy products. From a nutritional and health perspective, mare milk is considered more beneficial than cow’s milk. Chemically, mare milk closely resembles human milk [1,2,3]. It is rich in nutrients that play a role in human metabolism and helps to regulate physiological functions, enhance immunity, and prevent diseases [4]. Unsaturated and low-molecular-weight fatty acids in mare milk can prevent hypercholesterolemia and atherosclerosis and have therapeutic effects on conditions such as tuberculosis, emphysema, and chronic gastroenteritis [5]. Mare milk significantly contributes to human nutrition [6]. When breast milk is unavailable, mare milk, with its composition similar to human milk, serves as an excellent nutritional source for newborns. Traditionally, cow’s milk has been used as a substitute for human milk in infant nutrition. However, cow’s milk differs significantly from human milk in terms of its nutrient content, and the absorption rates of vitamins and minerals differ, potentially affecting infant health. Mare milk is more suitable as a substitute for human milk than cow’s milk [7].

Most bioactive peptides are usually obtained through the deliberate and controlled hydrolysis of proteins using specific proteases or microorganisms, which results in hydrolysates with excellent biological activities [8]. Native amino acid sequences and peptides generated during enzymolysis can be functionally active. Bioactive peptides demonstrate antioxidant, antithrombotic, antimicrobial, hypotensive, immunomodulatory, opioid, and other biological effects, which have curative or preventive impacts on the pathogenesis of several diseases [9,10,11,12].

Once released, these peptides have been proven to affect the neural, cardiovascular, endocrine, and immune systems in our body by exhibiting biological functions such as opioid, antihypertensive, anti-inflammatory, antimicrobial, antioxidant, antithrombotic, immunomodulatory, and osteoanabolic activities [13,14,15,16]. In the existing research, there are still relatively few studies on the bioactive peptides in mare milk. There may be several reasons for this. First, the nutritional value of mare milk has not been fully understood by people, so mare milk has not yet come into the public eye. Second, although nonbovine milk plays an important role in human health, mare milk is often overlooked. By contrast, research on sheep’s milk has already delved deeper than that on mare milk, and the nutritional components of mare milk still need to be explored.

This study aimed to systematically investigate the differences in potential bioactive peptides in mare milk at various temperatures and to identify the specific peptide sequences that exhibit various bioactive functions. This research provides a theoretical foundation for the development of peptides with different bioactivities as potential functional foods.

## 2. Materials and Methods

### 2.1. Milk Samples

The samples were collected from the Nanshan pasture located in the Xinjiang Uygur Autonomous Region of China, where the mares grazed freely. The grassland of Nanshan Ranch belongs to alpine grasslands, and there was no feed and feed residue seen around the mare pens. The climate of Nanshan Ranch belongs to the subtropical mountain monsoon humid climate, with no scorching heat in the summer and no severe cold in the winter, which is suitable for the growth of pasture grasses and is suitable for the feeding and living of mares. All mares had unrestricted access to food and water and were milked three times daily at designated time points. Samples were collected at 2 p.m., promptly removed from the sampling box containing ice packs, and transported to the laboratory for immediate storage in a −80 °C freezer (Vertical Midea Ultra-low Temperature Refrigerator MD-86L58, Midea Group Co., Hefei, China).

### 2.2. Extraction of Whey Proteins

Fresh mare milk was centrifuged at 5000× *g* for 20 min, and this process was performed three times to remove fat for degreasing. Skim milk and 2 M of sodium acetate were mixed with 10% acetic acid to adjust the pH to 4.6. The sample was then centrifuged at 12,000× *g* for 60 min to remove caseins. The treated whey proteins were heated at 65 °C for 30 min, 72 °C for 15 min, 83 °C for 10 min, and 95 °C for 5 min, with the untreated group used as a control [17]. The supernatant (whey protein) was collected and stored in a refrigerator at −80 °C.

### 2.3. Assessment of Protein Purity Using SDS-PAGE

The purity of the protein was assessed using SDS-PAGE [18]. About 5 mL of distilled water was mixed with 5.8 mL of 30% acrylamide (29:1, 30% solution. Ranjeko Technology Co., Hefei, China), 2.5 mL of 1.5 M of Tris-HCl (pH 8.8, Ranjeko Technology Co., Hefei, China), 100 µL of 10% SDS, 100 µL of 10% ammonium persulfate, and 20 µL of tetramethylethylendiamine (PAGE gel accelerator, Shanghai Zeye Biotechnology Co., Shanghai, China) in a 50 mL centrifuge tube to prepare the separation gel. About 2.84 mL of distilled water was mixed with 2.84 mL of 30% acrylamide, 100 µL of 10% SDS, 75 µL of 10% ammonium persulfate, 1.25 mL of 4× concentrating gel, and 10 µL of tetramethylethylenediamine in a 50 mL centrifuge tube to prepare the concentrating gel.

Ten micrograms of each sample were added to an appropriate amount of a 5× loading buffer (5× Tris-glycine electrophoresis buffer, Ranjeko Technology Co., Hefei, China), heated in a boiling water bath for 5 min, and then subjected to SDS-PAGE electrophoresis. Finally, Coomassie Brilliant Blue R-250 [19] (Beijing Dingguo Changsheng Biotechnology Co., Beijinng, China) staining was performed, and a decolorizing solution (ethanol:acetic acid:water = 1:2:17) was used [20].

### 2.4. Protein Extraction and Peptide Enzymolysis

An appropriate amount of UA (7 M, 50 mM of Tris-HCl, and pH 7.8, Beijing Prilosec Gene Technology Co., Beijing, China) lysate was added to each sample to extract the protein, and the protein was quantified using the Coomassie Brilliant Blue method. The protein extracted from the lysate was then enzymatically digested by trypsin and pepsin. The specific methods are as follows:

First, the pepsin was adjusted to the optimal pH of 3.0. The pH was adjusted using 0.2 mol/L of acetic acid (analytically pure, Aladdin Reagent (Tianjin Xinbute Chemical Co., Tianjing, China) and 0.5 mol/L of sodium hydroxide (analytically pure, Aladdin Reagent (Shanghai) Co., Ltd., Shanghai, China). The sample was enzymatically digested for 4 h. Second, the pH of the solution was adjusted to 8.0 with acetic acid, and the sample was enzymatically digested with trypsin for 8 h. After enzymolysis, the peptide segments were desorbed using a C_18_ (Thermo Scientific EASY column, Commonwealth of Massachusetts, 10 cm, ID 75 μm, 3 μm. Thermo Fisher Scientific. Waltham, MA, USA) cartridge; lyophilized; and redissolved in 20 μL of a 0.1% formic acid solution. The peptide concentration of the sample was determined using OD_280_.

### 2.5. Peptide Treatment

For the peptide samples, ultrafiltration was performed using a 5 kDa ultrafiltration membrane (Shanghai Yuanye Biotechnology Co., Shanghai, China) and a C_18_ cartridge. The peptide was desalted, lyophilized, and redissolved in 20 μL of a 0.1% formic acid solution. The peptide concentration was determined using OD_280_.

### 2.6. LC–MS/MS Data Acquisition

Each sample was separated using the HPLC liquid phase system (Easy nLC) at a nanoliter flow rate. Buffer A was a 0.1% formic acid aqueous solution, and Buffer B was a 0.1% acetonitrile aqueous solution (84% acetonitrile, analytically pure, Aladdin Reagent (Shanghai) Co., Ltd., Shanghai, China).The column was balanced with 95% of Buffer A, and the sample was loaded from the automatic injector onto the column. The samples were separated using chromatography and analyzed using a mass spectrometer. The detection method used positive ions, with a scanning range of 300–1800 *m*/*z*. The primary mass spectrometry resolution was 60,000 at 200 *m*/*z*, the Automatic Gain Control target was 1 × 10^6^, and the maximum injection time was 50 ms. The dynamic exclusion time was 30.0 s.

### 2.7. Prediction of Bioactive Peptides

#### 2.7.1. Prediction of ACE Inhibitory Peptide Binding

The Angiotensin-I-Converting Enzyme (ACE) Inhibitory Peptide (AHT Peptide Fusion) Predictive Modeling was used to predict ACE inhibitory peptides. It was predicted that ACE inhibitory peptides with lengths of 2 to 15 amino acid residues and different activity distributions showed excellent performance (accuracy > 0.9). Among 12 machine learning (ML) algorithms, Transformer outperforms 11 other models and performs best in predicting short and medium peptides. In an external dataset, AHTPeptideFusion, a fusion of Transformer and Random Forest (RF), showed excellent performance in predicting ACE-I inhibitory peptides with lengths ranging from 2~15 amino acid residues and varying activity distributions (accuracy > 0.9) [21].

#### 2.7.2. Prediction of Glucosidase Inhibitory Peptide Binding

The data of 208 glycosidase inhibitory peptides were collected from the literature, and the IC_50_ threshold of glycosidase inhibitory activity was defined as 10 mM. When the IC_50_ was less than 10 mM, it was considered to have inhibitory activity, and when the IC_50_ was more than 10 mM, it was considered to have low inhibitory activity or no inhibitory activity. The training set consisted of 69 highly inhibitory peptides and 96 peptides with low or no inhibitory activity; the test set consisted of 17 highly inhibitory peptides and 25 peptides with low or no inhibitory activity.

**Figure 1:**
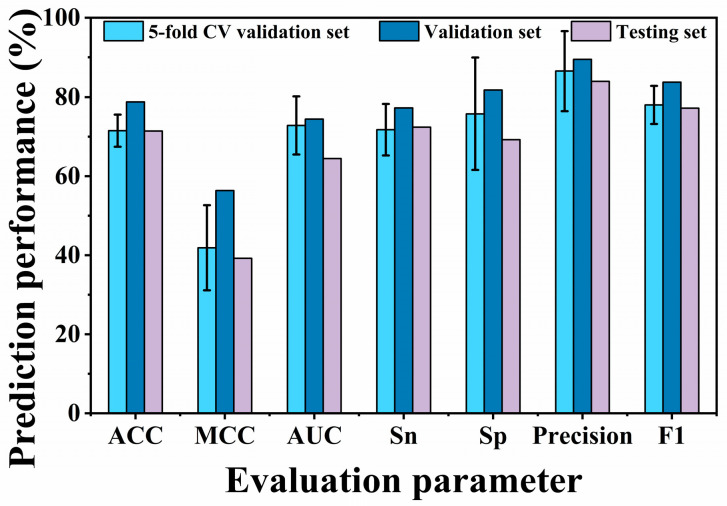
Model performance of LBSPP in the training set and test set.

LBSPP model building is based on the protein language model ESM-2 and the convolutional neural network model (CNN), which is subjected to a five-fold cross-validation in the training set, followed by testing in the test set. The LBSPP model was used to predict ACE activity. Data on 208 glucosidase inhibitory peptides were collected from the literature, and the IC_50_ threshold of glucosidase inhibitory activity was defined as 10 mM (Figure 1).

#### 2.7.3. Prediction of In Vitro Antioxidant Modeling

The protein language model ESM-2 and the convolutional neural network model were used to predict antioxidant activity. Usage: based on the protein language model ESM-2 and the convolutional neural network model (CNN), a ten-fold cross-validation was performed on the training set, followed by testing on the test set.

## 3. Results

### 3.1. Assessment of Purity and Molecular Weight of Proteins Using SDS-PAGE

**Figure 2:**
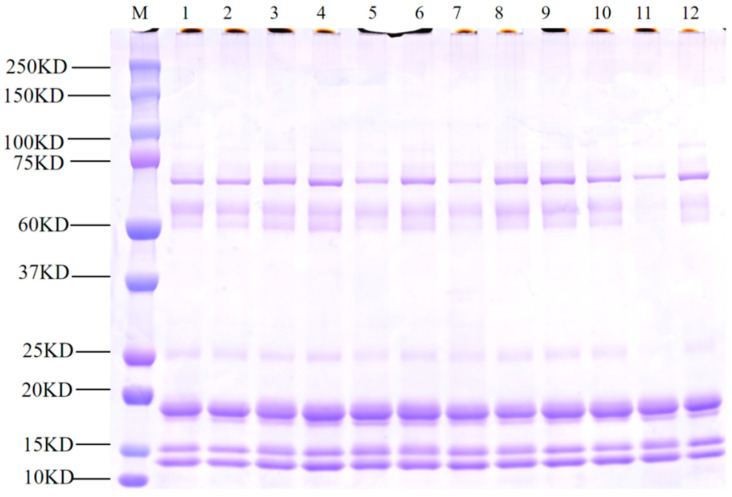
The electropherogram shows the whey proteins using SDS-PAGE. Lane M: standard protein markers (low-range rainbow marker, Lanes 1–12 proteins from mare milk).

The electropherogram shows bands due to the proteins, indicating the purity and molecular weight of the mare milk. Lane M was a standard protein marker (low-range rainbow marker). The molecular weights of the isolated proteins from the mare milk were between 15 and 20 kDa (Figure 2).

### 3.2. Amino Acid Changes During Heating

**Figure 3:**
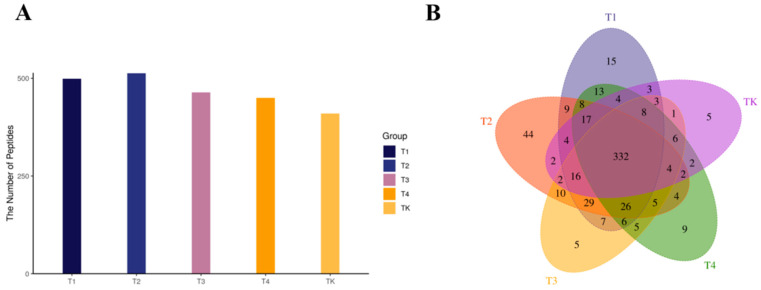
(**A**) The vertical coordinate represents the number of peptides identified between all sample groups. The horizontal coordinate is the group name of the test group. (**B**) A Venn diagram of peptides identified between all sample groups. Note: it is important to note that the change in peptide content here is relative content, not absolute content.

In the following steps, we analyzed endogenous peptide profiles with mare whey protein samples heated at 65, 72, 83, and 95 °C and used the untreated group as a blank control using LC–MS/MS combined with a search of the PEAKS X+ database. An overall analysis of all samples identified 2341 unique peptides corresponding to 14 different proteins. A total of 2341 active peptides were identified in 15 samples from five test groups. Among them, 500 active peptides were identified in group T1, 514 in group T2, 465 in group T3, 451 in group T4, and 411 in group TK (Figure 3A). There were 332 active peptides common to all five test groups. There were 15 specific active peptides in group T1, 44 specific active peptides in group T2, five specific active peptides in group T3, nine specific active peptides in group T4, and five specific active peptides in group TK (Figure 3B). The other 935 peptides had repeats in multiple groups.

### 3.3. Effect of Temperature on Molecular Weight and Amino Acid Composition of Potential Polypeptides

Each amino acid has different physical and chemical properties and functions, and the difference in amino acid composition determines the diverse physical and chemical properties and functions of peptides. The antioxidative and functional properties of peptides have a direct relationship with the components of amino acids and their structural and hydrophobic properties.

**Figure 4:**
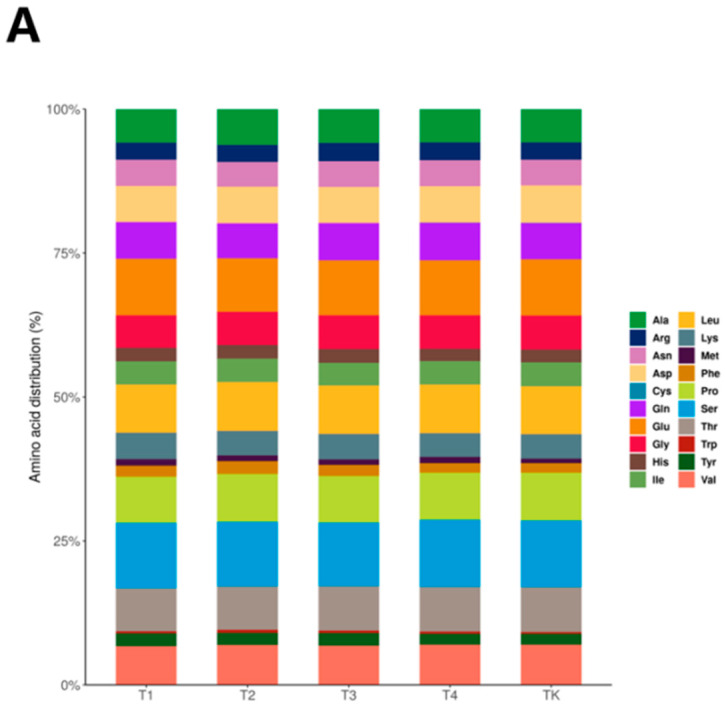

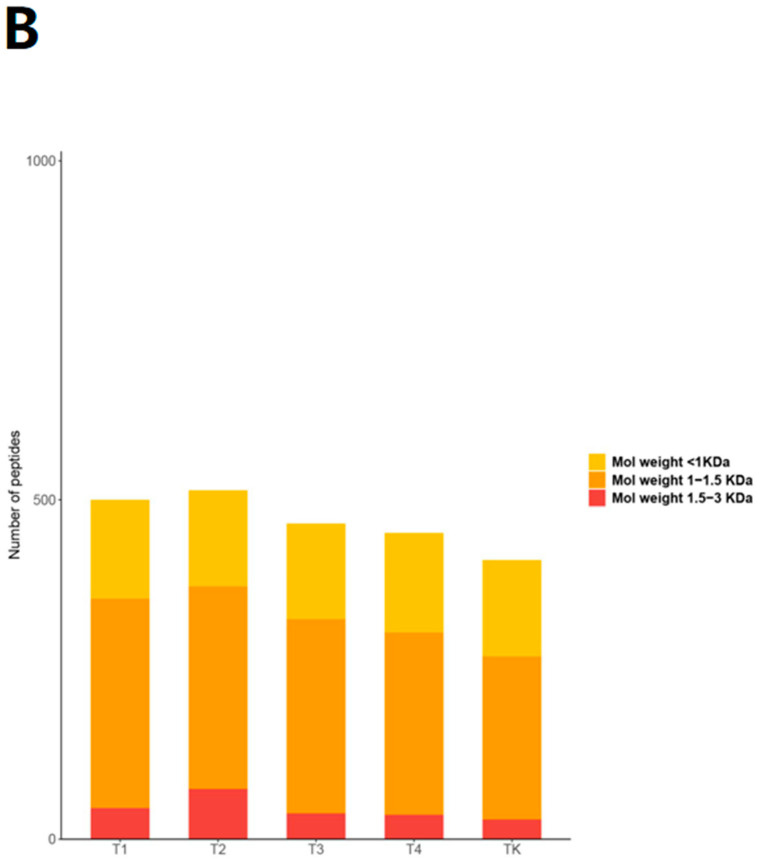

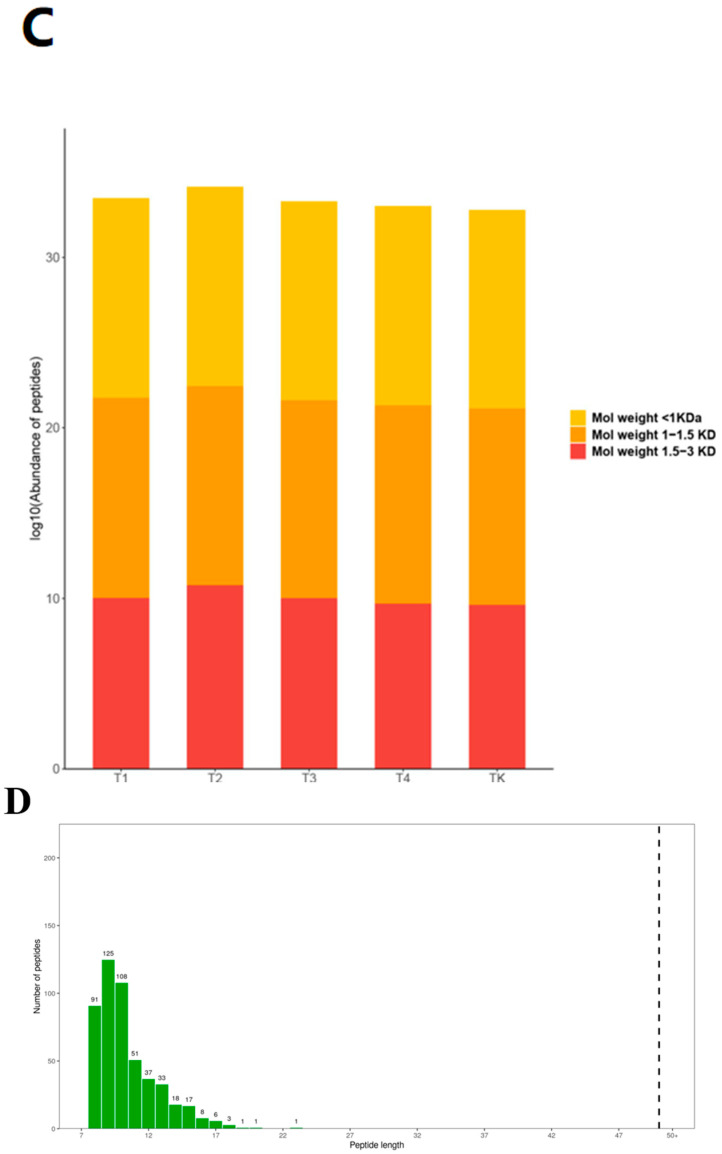

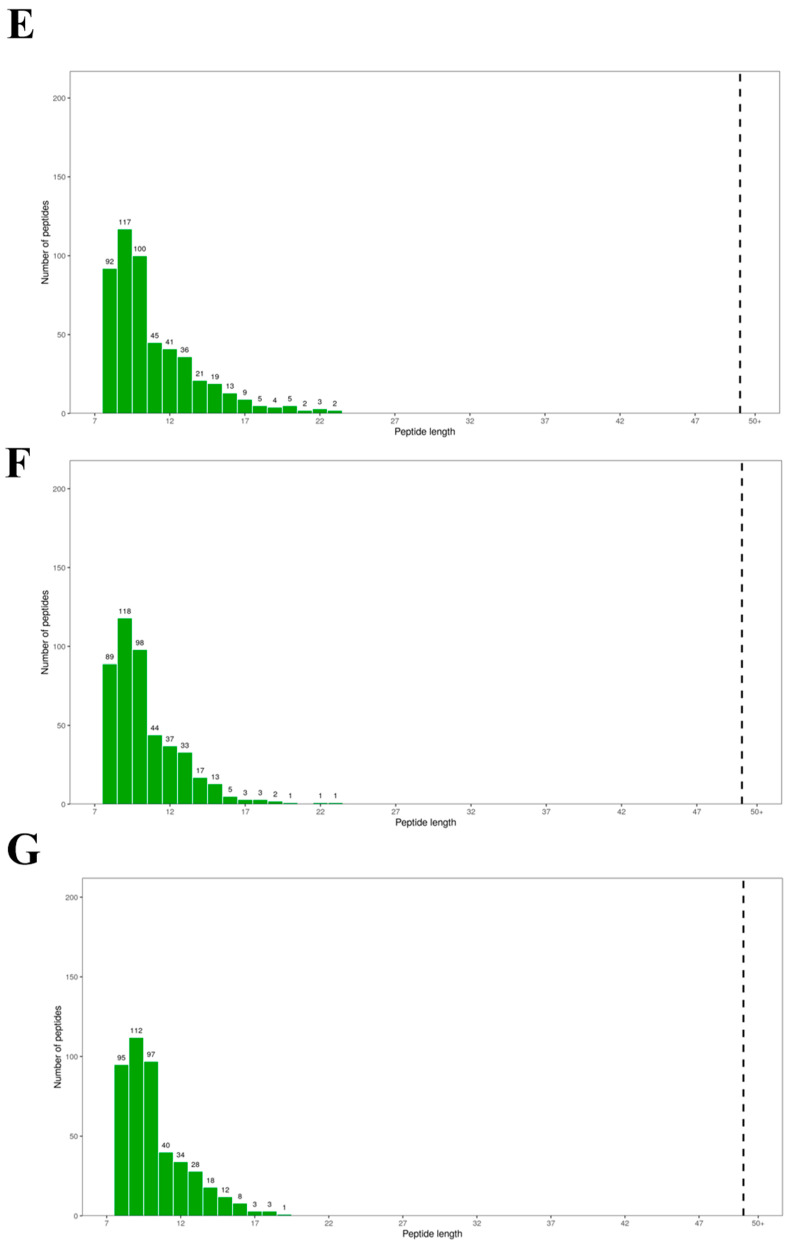

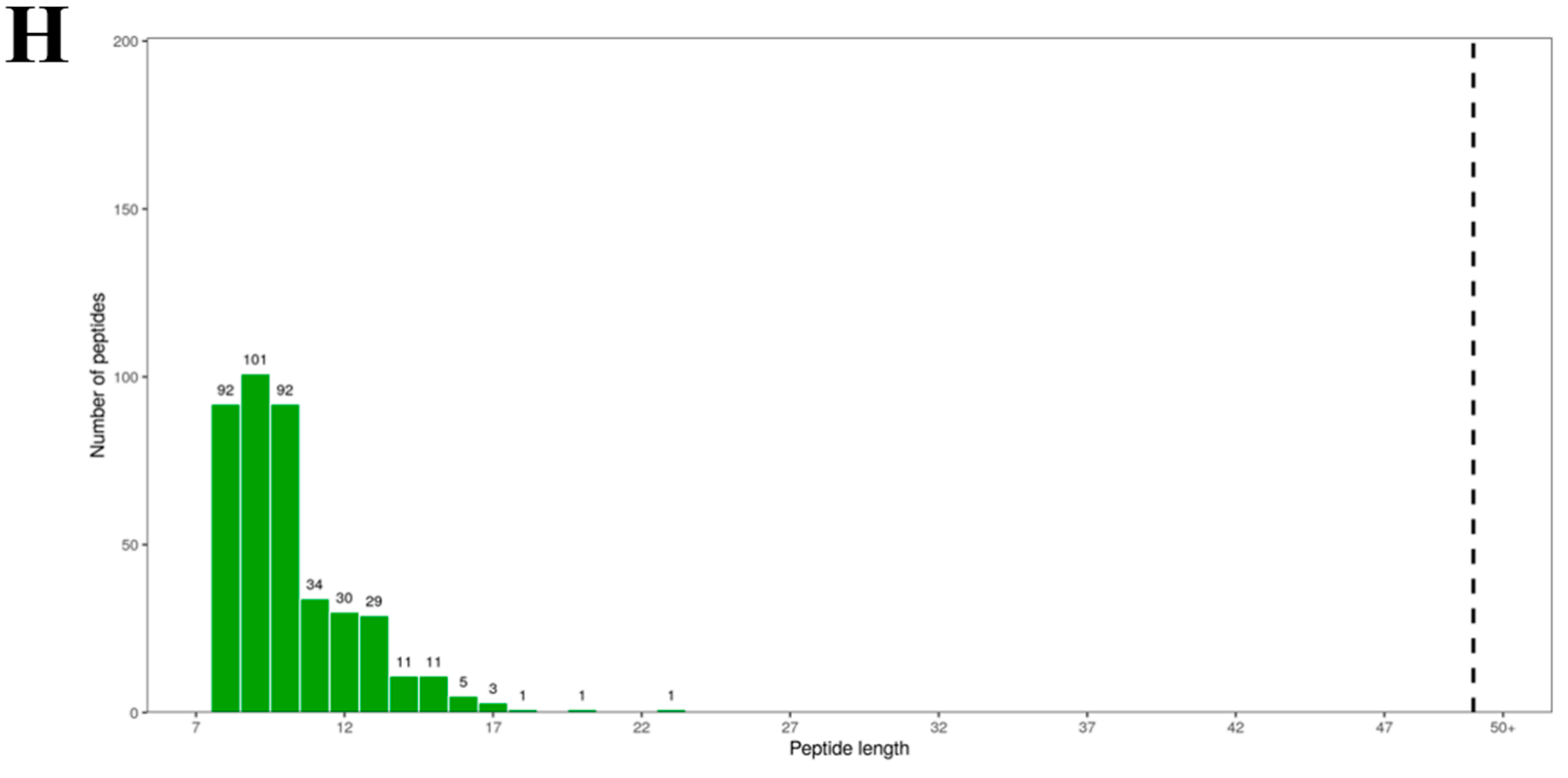
(**A**) The horizontal coordinate represents the group name, the vertical axis represents the percentage of amino acids, and the color represents the 20 amino acids. (**B**) A statistical accumulation map of the number of polypeptides in different molecular weight intervals for each group of samples. (**C**) The abundances of peptides in different molecular weight ranges for each group of samples. The horizontal coordinate is the group name, and the vertical coordinate represents the peptide abundance (signal response strength) at different molecular weights. (**D**–**H**) The quantitative distribution of the length of identified polypeptides. Note: The collected milk was treated at different temperatures, including T1 (**D**), T2 (**E**), T3 (**F**), T4 (**G**), and TK (**H**). The peptide content here is relative, not absolute.

In order to understand the composition of amino acids in the samples and the differences between the groups, a Column Accumulation Chart was used to analyze the whole amino acid composition of the polypeptides. The composition of amino acids did not change significantly with an increase in temperature (Figure 4A).

The molecular weight and length of polypeptides can affect digestion and absorption in the body, the activation of enzyme systems, and the growth and fermentation of microorganisms. To analyze the distribution of the molecular weight and length of polypeptides between the sample groups and the similarities and differences in polypeptide spectra among different groups, a column accumulation diagram was used. The molecular weights of the peptides identified in the T1, T2, T3, T4, and TK samples were primarily in the range of 1–1.5 kDa, followed by some peptides in the range of <1 kDa, and a few peptides in the range of 1.5–3 kDa (Figure 4B). There was no significant difference in peptide abundance among T1, T2, T3, T4, and TK, indicating that temperature had no significant effect on peptide abundance (Figure 4C). Peptides composed of 2 to 10 amino acids are short peptides, and peptides composed of 10 to 50 amino acids are long peptides. The T1 group had 324 short peptides and 175 long peptides (Figure 4D). The T2 group had 309 short peptides and 205 long peptides (Figure 4E). The T3 group had 305 short peptides and 160 long peptides (Figure 4F). The T4 group had 298 short peptides and 147 long peptides (Figure 4G). The TK group had 285 short peptides and 126 long peptides (Figure 4H).

### 3.4. Gene Ontology (GO) Analysis and KEGG Pathway Analysis

**Figure 5:**
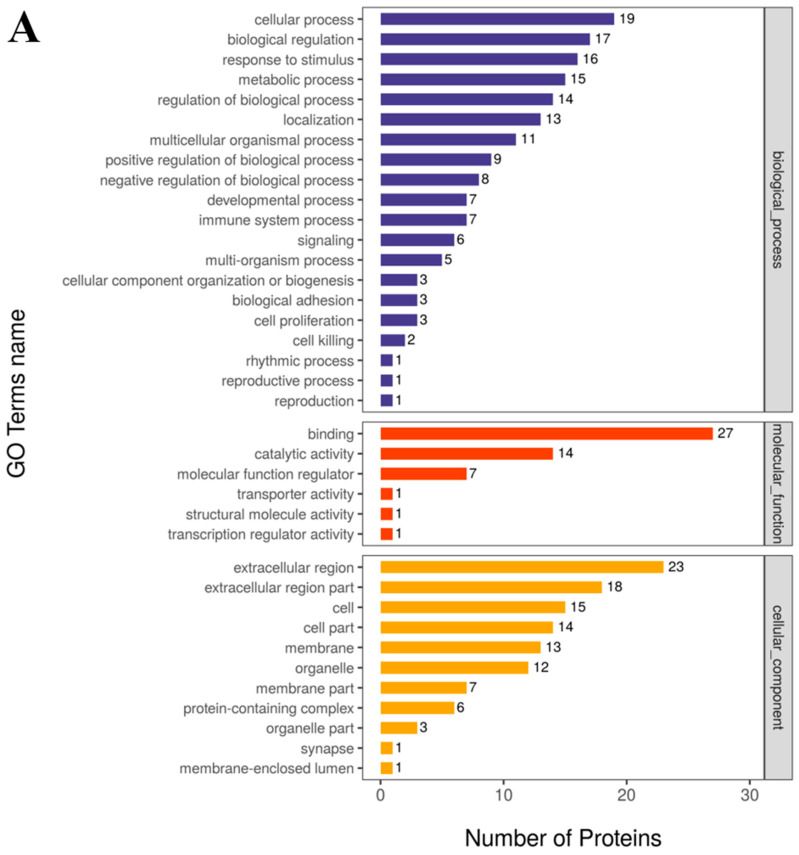

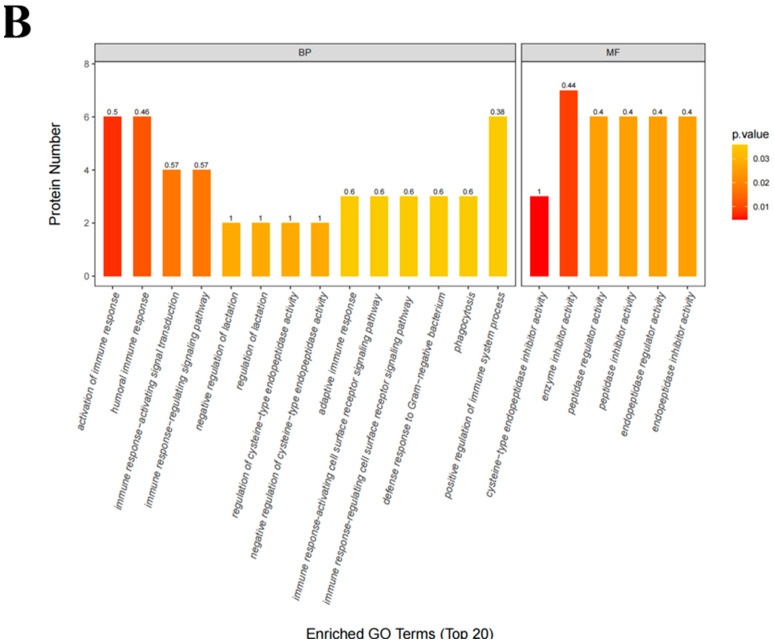
The identified protein functions were annotated by a Gene Ontology (GO) analysis (**A**) and a metabolic pathway analysis (**B**). Note: The horizontal ordinate is the number of proteins, whereas the vertical coordinate is the annotated GO (**A**) and KEGG (**B**) entries. Because of the large number of GO annotation results, (**A**) only shows the top 10 results in each category. The dark blue bar represents a biological process. The orange–red bar means represents molecular function. The yellow bar represents a cellular component.

In total, 2341 active peptides were identified in 15 samples from the five test groups. The functions of the identified proteins were annotated using a GO analysis and metabolic pathway analysis (Figure 5A). The GO analysis revealed that these proteins were mainly related to biological processes (BPs), such as a cellular process, biological regulation, a reaction to a stimulus, a metabolic process, the moulation of a biological process, and localization; cellular components (CCs), such as the extracellular region, a part of the extracellular region, the cell, a part of the cell, and the membrane; and molecular functions (MFs), such as binding, catalytic activity, and increased molecular function(Figure 5B).

### 3.5. The Difference in Potential Polypeptide Expression Under Different Heat Treatment Conditions

**Figure 6:**
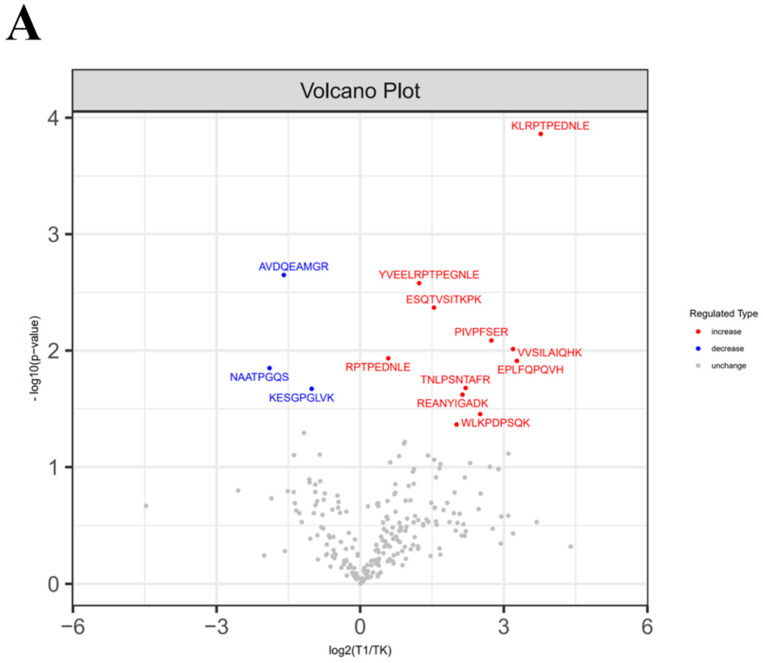

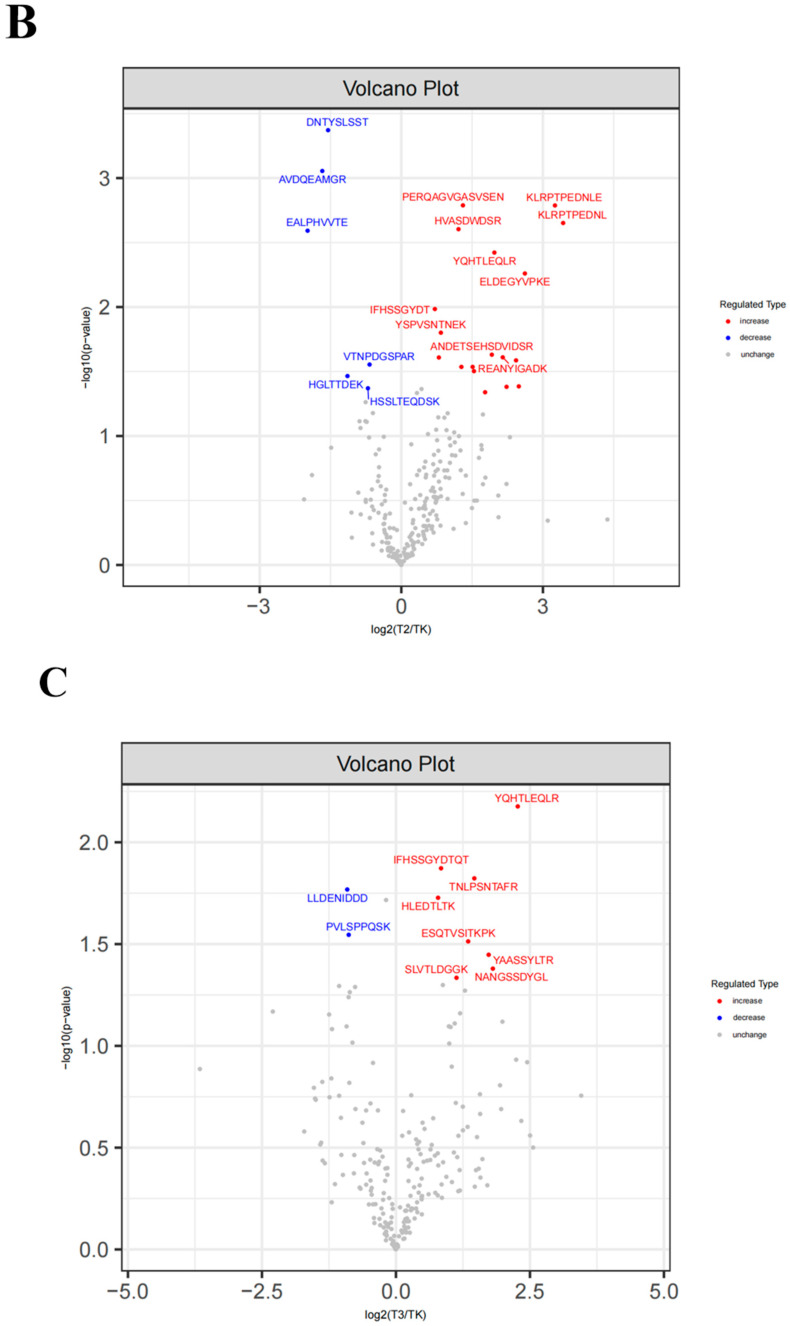

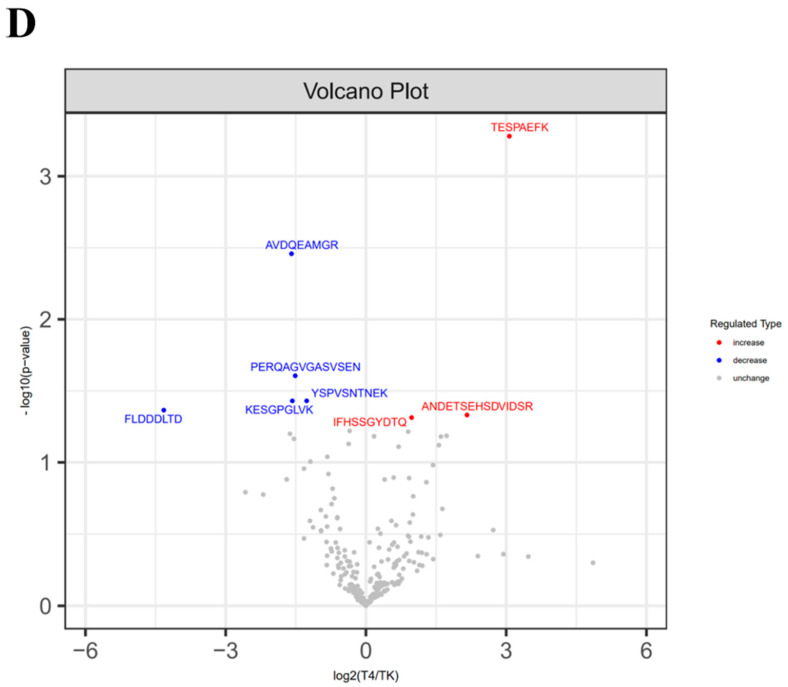
Volcano plots of bioactive peptides in mare milk at different temperature stages, utilizing bioinformatic and polypeptidomic analyses. Note: Milk samples were collected from Nanshan mares at four different temperature stages, 65 (**A**), 72 (**B**), 83 (**C**), and 90 °C (**D**), and 0 °C (TK). Note: Additional milk samples were collected from Nanshan mares at four different temperature stages, designated as T1VSTK (**A**), T2VSTK (**B**), T3VSTK (**C**), and T4VSTK (**D**). Change here refers to relative content change, not absolute content change.

The number of identified bioactive peptides (BAPs) in mare milk at different temperature stages is shown in Table 1. The volcano plots of BAPs in mare milk at the temperatures of 65, 72, 83, and 95 °C are shown in Figure 6. The differences in BAPs in mare milk between the different temperature stages are shown in Table 1. The subcellular localization of the BAPs is shown in Figure 6.

**Figure 7:**
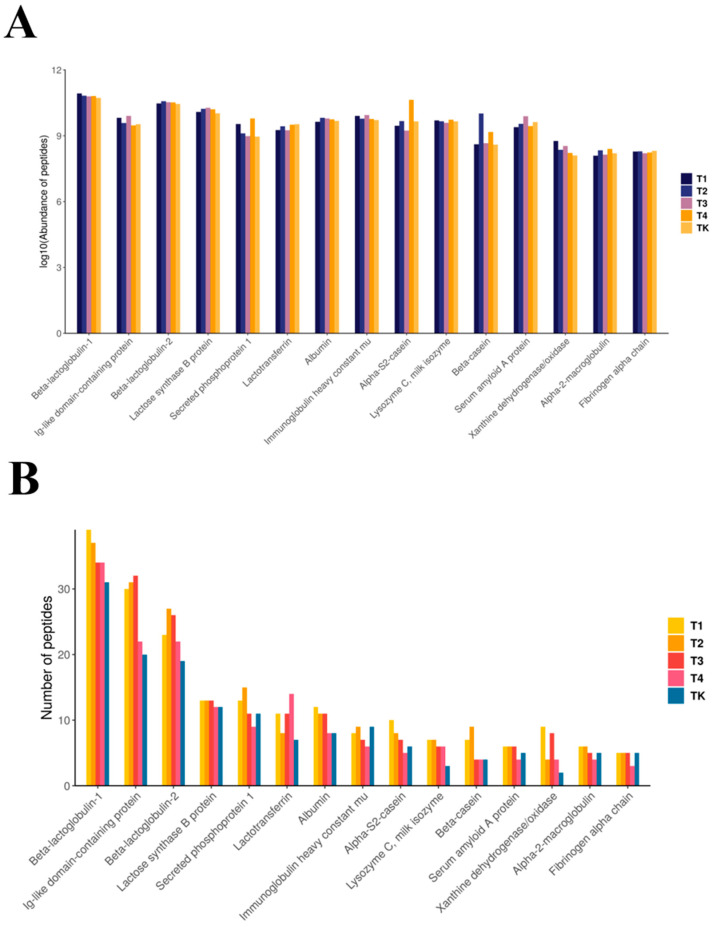
(**A**) Histogram of the number of proteins corresponding to polypeptides. (Description: the horizontal axis represents the protein name (top 15), and the vertical axis represents the number of peptides). (**B**) Histogram of proteins corresponding to polypeptide abundance. (Description: the horizontal axis represents the protein name (top 15), and the vertical axis represents the peptide abundance, indicated by signal response strength, under different molecular weights).

**Figure 8:**
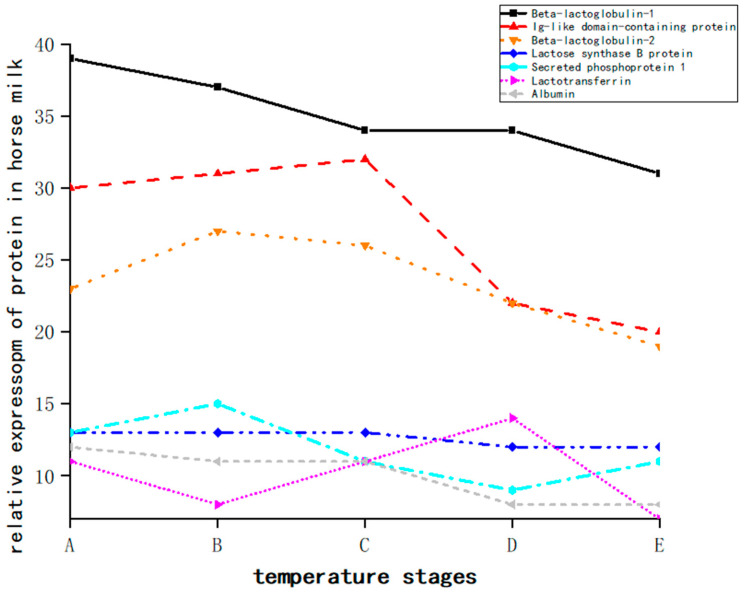
The expression levels of each protein of whey protein at different temperature stages. Note: mare whey protein samples were treated at temperatures of 65 (A), 72 (B), 83 (C), 95 °C (D), and (E) stands for a blank group at the five different heating stages.

The major contributing proteins of peptides were analyzed further, and the difference in the contributions of major contributing proteins in different groups were compared. The results show that the identified active peptides mainly came from β-lactoglobulin-1, an Ig-like domain-containing protein, and β-lactoglobulin-2 (Figure 7). The expression abundance of all bioactive peptides in the experimental groups (groups 1, 2, and 3) was higher than that in the control group, except for T4 in the experimental groups. With an increase in temperature, the expression quantity of most bioactive peptides gradually decreased, so it can be concluded that about 72 °C is the optimal temperature for bioactive peptides. With an increase in temperature, the relative expression of β-lactoglobulin-2 gradually decreased, and the relative expression of the Ig-like domain-containing protein gradually increased, until the relative expression of immunoglobulin reached the maximum at 83 °C and plunged to 95 °C (Figure 8).

### 3.6. Metabolic Pathway Analysis of Potential BAPs

**Figure 9:**
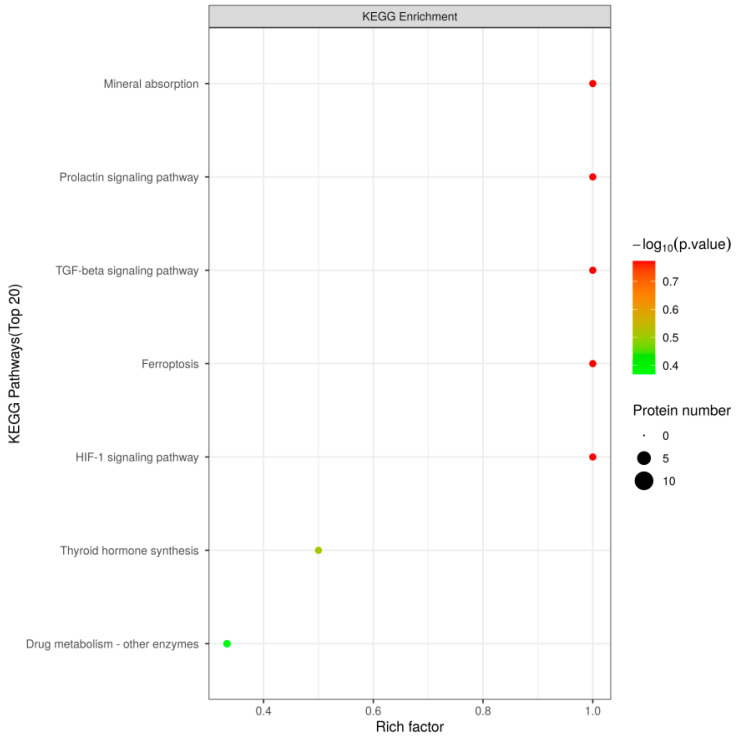
The KEGG pathway of the polypeptide-corresponding protein.

The bioactive peptides identified through heat treatment can be categorized into CC, MF, and BP based on their functions. The GO analysis showed that increased peptide expression was primarily related to BPs (Figure 9), such as mineral absorption, the prolactin signaling pathway, the TGF-beta signaling pathway, and ferroptosis. By contrast, a decrease in peptide expression was mainly related to MFs and BPs, such as other drug-metabolizing enzymes and thyroid hormone synthesis (Table 2).

## 4. Discussion

Mare milk is a traditionally consumed food in several regions of the world due to its high nutritional value and potential beneficial effects on human health. In Russia and Asia, and to a lesser extent, in Europe, mare milk (and its fermented version, kumis) has been used as a health-promoting food for the treatment of chronic hepatitis, peptic ulcers, tuberculosis, bronchitis, asthma, anemia, nephritis, diarrhea, and gastritis, among others [22,23]. Compared with other mammals, mare milk is low in protein abundance but rich in whey proteins, similar to donkey’s and human milk. In fact, this protein fraction accounts for approximately 35% (by weight), which significantly differs from ruminant milk, containing about 80% caseins and only 20% whey proteins [24,25]. However, little research has been conducted on the potential presence of bioactive peptides in mare whey proteins, especially under heat treatment. This is the first study to investigate potential bioactive peptides in mare milk under heat treatment and a biofamily of hydrolysis products using peptidomics and bioinformatics.

### 4.1. Effect of Heat Treatment on the Composition of Mare Whey Proteins

Bioactive peptides in mare milk have attracted more and more researchers’ interest due to their diverse bioactivities. The most notable factors affecting these bioactive properties include chain length and amino acid sequence, with the amino acids at the termini being the most relevant for many reactions [26,27,28]. Studies have shown that heat treatment can increase the content of Glu, Asp, Met, and Cys in milk to varying degrees, increasing the frankincense flavor and improving the flavor of the product [29]. This is consistent with the results of this study. The results show that the content of Met increased at 65 °C, 30 min; 72 °C, 15 min; 83 °C, 10 min; and 95 °C, 5 min. The content of Ala increased under heat treatment at 72 °C. The content of Arg increased at 72 °C and 83 °C.

Amino acids can be divided into hydrophobic and hydrophilic amino acids; hydrophobic amino acids (isoleucine, leucine, methionine, phenylalanine, tyrosine, threonine, valine, alanine, glycine, proline, and serine); amino acids with the ability to chelate transition metals (histidine, aspartic acid/aspartate, and glutamate/glutamine); and amino acids with the ability to provide electrons/hydrogen (glutamate/glutamine and thionine) [30]. Arginine, lysine, and histidine, that is, amino acids that have a positive charge and are associated with antimicrobial activity, are also present in all grades [31]. Studies have shown that during the heating process of raw milk, the protein will undergo enzymolysis or the chemical bond between amino acids will be broken, so that the amino acids themselves will be cracked, or the Maillard reaction will occur with the sugars in the milk, resulting in a change in the content of free amino acids [32,33]. The α-lactalbumin concentration was higher in equine dairy animals at 0.27 g/mL. This is consistent with the results of this study, in which the content of some amino acids changed with an increase in heat treatment conditions.

### 4.2. Effect of Heat Treatment on Potential Peptidomics

The α-lactalbumin (α-LA) concentration in mare milk, donkey’s milk, and camel’s milk is high [34]. These milks contain lysozyme, lactoferrin, and other active ingredients, which possess antibacterial and antiviral properties. The extract obtained from mare milk is rich in collagen and calcium and is known as the “breast milk factor”. It has a certain effect on conditions such as pharyngitis, influenza, respiratory tract inflammation, urinary system inflammation, and ear inflammation [35].

With an increase in temperature, the percentages of β-lactoglobulin-1 and β-lactoglobulin-2 first decreased and then increased. At 95 °C, the percentages of β-lactoglobulin-1 and β-lactoglobulin-2 reached the highest values of 16.19% and 10.49%, respectively. The proportion of immunoglobulin domain proteins first increased and then decreased, and the proportion of immunoglobulin domain proteins reached the highest value of 12.75% at 83 °C. This is consistent with Arrutia’s findings that the heat treatment of proteins before enzymatic hydrolysis can alter the distribution of peptides released during hydrolysis and that heat treatment significantly increases the degree of hydrolysis and the number of peptides obtained [36]. Most of the peptides found by Leite in heat-treated cow, sheep, and goat milk are derived from β-casein and αs1-casein [37], which match the hydrolytic properties of cathepsin D and plasminase. This is inconsistent with the results of this study, which found that the active peptides present were mainly derived from β-lactoglobulin and immunoglobulin and immunoglobulin domain proteins. Possibly because of differences in milk varieties or treatment problems, Leite only used two temperatures, 63 °C and 85 °C. In this study, there were four treatment temperatures, 65, 72, 83, and 95 °C. The use of a mild heat treatment, such as 65 °C/30 min, preserved the activity of the studied natural proteolytic enzymes in ruminant milk and produced new potentially bioactive peptides. Abeer heated camel milk at 80 °C for 30, 60, 90, and 120 min. With an extension in heat treatment time, the ash content of camel milk remained basically unchanged, and the α-S2 casein changed. This is consistent with the results of this study, which found that heat treatment can lead to changes in the content of certain proteins [38].

### 4.3. Effects of Heat Treatment on Metabolic Pathways of Potential Active Peptides

In this study, 47 different peptides were identified under various temperature treatment conditions, among which 29 peptides were increased, and 18 peptides were increased, such as those involved in mineral absorption, the prolactin signaling pathway, the TGF-beta signaling pathway, ferroptosis, and the HIF-1 signaling pathway. Immunoglobulin domain proteins were the primary proteins expressed by differentially expressed polypeptides. The proteins corresponding to the polypeptides with a change magnitude greater than 5 mainly included β-lactoglobulin-1, the Ig-like domain-containing protein, β-casein, secreted phosphoprotein 1, and albumin. Among these, eight polypeptide-expressing proteins were located in the immunoglobulin domain. This indicates that mare milk has unique advantages in increased immune activity.

The metabolic pathways that decrease polypeptides include thyroid hormone synthesis and drug metabolism involving other enzymes [39]. P450 primarily participates in drug metabolism, and it is not the only enzyme involved in the metabolism of drugs and other heterologous biomass chemicals. All enzymes catalyze the binding of chemicals to low-molecular-weight compounds already present in cells, or they catalyze hydrolysis reactions. These enzymes are often collectively referred to as phase II reactions, with P450 and other oxidations being phase I [40].

### 4.4. The Biological Activities of Milk Proteins in Mare Milk

The sequences of 681 peptides were determined through computer simulations. The results show that the antioxidant activities of ABTS, DPPH, FRAP, and ORAC were determined, with the corresponding numbers of peptides being 513, 616, 590, and 641, respectively. The number of peptides with glucosidase inhibitory activity and ACE inhibitory activity were 616 and 583, respectively, and among these, 457 peptides had multiple biological activities.

The length, number, and chemical properties of the polypeptide, the amino acid sequence, and the composition associated with the spatial structure determine the activity of the peptide released from the original protein [41,42,43,44]. Although the correlation between the structural and functional properties of peptides has not been clearly derived, many of them share common constituent characteristics, such as the presence of average amino acids in the molecule, hydrophobic amino acids, proline, lysine, or arginine [44], all of which have been discussed in this study.

Compared with the other four temperatures, the difference multiples of the active peptides identified at the T1 and T2 temperatures were greater than 5, and there were five peptides. The proteins corresponding to these active peptides are mainly β-lactoglobulin (β-LG) and immunoglobulin domain proteins. β-LG is a globular protein, with a molecular mass of 18.3 kDa, that belongs to the lipocalin family [45].

β-LG is the major whey protein in the milk of ruminants, such as cows and goats [46]. β-LG has also been found in the milk of monogastric animals, but it is absent in human milk, which has α-LA as the dominant whey protein [47]. Heating temperature, pH, concentration, and ionic strength can affect β-LG’s physiological conditions [48]. This is consistent with the results of this study. Heat treatment changes the content of β-LG, and the proportion of β-LG first decreases and then increases with an increase in temperature.

Heat treatment can lead to reduced levels of water-soluble vitamins such as vitamin B12, vitamin E, vitamin C, folic acid, and riboflavin [49]. In the heat treatment process [50], this will lead to the loss of lysine in milk and an increase in free fatty acid content and will also lead to the degeneration or change in quantity of various natural enzymes and microbial metabolism in milk [51].

Whey proteins are very susceptible to thermal denaturation, and sensitivity depends on the structural chemistry of the protein. Immunoglobulin is the most sensitive whey protein, followed by bovine serum albumin, β-LG, α-LA, and protein glycopeptides, which are not affected by heat [52]. Compared with β-lactoglobulin-l and α-LA, the degeneration of immunoglobulin has less interaction with other proteins, whereas the degeneration of whey proteins in milk is mainly caused by β-LG degeneration, accounting for about 50% of whey proteins, which is consistent with the results of this study, which found that the proportion of β-LG first decreases and then increases with an increase in temperature [53].

Numerous beneficial properties for human health have been described for milk and dairy products, making them significant sources of bioactive molecules [54]. Peptides released from milk proteins, in particular, have been shown to perform a variety of physiological functions, including antimicrobial, antioxidant, antithrombotic, anti-inflammatory, and immunomodulatory functions. Two effective DPP-IV inhibitory peptides, LPVP and MPVQA, were identified in the protein hydrolysate of camel milk, and the half-inhibitory concentration of DPP-IV was <100 µM. The identified DPP-IV inhibitory peptides, such as LPVP, LPLPL, and LPVPQ, are active peptides specific to camel milk and are not currently found in cow milk. This may be related to the digestibility by gastrointestinal enzymes in camels and cattle [55]. A sum of 5162 and 940 endogenous peptides from 258 forerunner proteins were distinguished in human colostrum and mature milk by a dataset search. A total of 2446 differentially expressed endogenous peptides with various biological functions were found among these peptides. Studies have shown that CCs, BPs, and MFs are related with these maternal proteins [56].

Polypeptides can act as regulatory molecules for DNA. They act as “switches” for DNA, controlling which genes should be expressed and thus affecting life activities such as cell growth, differentiation, and metabolism. Short peptides of less than eight amino acids were used in a study to treat mice with type I diabetes and were found to control insulin levels and restore blood sugar levels [57]. Short peptides consisting of 2–7 amino acid residues can enter the nucleus of cells and interact with nucleosomes, histones, and DNA. DNA–peptide interactions, including sequence recognition in gene promoters, are important for template-directed synthetic reactions, replication, transcription, and repair [58].

In contrast to raw milk, heated mare milk has been found to release a higher number and content of peptides during enzymatic digestion. However, studies on the digestion of bioactive peptides derived from mare milk proteins in the gastrointestinal tract are limited; it is still necessary to combine the effects of heat treatment on their release and absorption for further discussion.

So far, research and databases on bioactive peptides have mainly focused on cow’s milk and goat’s milk. The sequence of mare milk exhibits high homology with cow and goat milk, which makes it possible to utilize bioactive peptide databases based on cow’s milk data. Consequently, comparing active peptides in mare milk to ascertain their potential biological activities is a promising strategy, particularly when sequence matching is achieved to 100 percent. However, to accurately assess biological activities, sequence homology alone is insufficient. In order to confirm the functional properties and biological activities of the identified peptides from various milk sources, experimental confirmation through in vitro and in vivo experiments is necessary.

## 5. Conclusions

This is the first comprehensive analysis of changes in the entire peptide set in mare whey proteins under different heat treatment conditions, revealing changes in milk protein composition and peptide profile with temperature changes. According to the different heat treatment conditions, bioactive peptides showed different biological characteristics. According to the test results, we can see that the best heat treatment condition was 72 °C, the most active peptides were identified at 15 min, and increased peptides were also the most prevalent. A next step could be to determine whether and how these peptides maintain their activity in the body, in particular, whether the content of these potentially active peptides changes after absorption in the gastrointestinal tract and what their mechanism of action is.

## Figures and Tables

**Table 1:** The number of differential BAPs in mare milk in different temperature stages.

Compared Sample	Number of Polypeptides	Relative Content	Number	Fold (>1.50)
T1VSTK	579	Increased	11	137
		Decreased	3	137
T2VSTK	672	Increased	10	86
		Decreased	6	151
T3VSTK	544	Increased	8	59
		Decreased	2	228
T4VSTK	530	Increased	3	56
		Decreased	5	156

**Table 2:** Analysis of the metabolic pathways of different polypeptides.

GO Class	Relative Content	Metabolic Pathway
BP	Increased	Mineral absorption
		Prolactin signaling pathway
		TGF-beta signaling pathway
		Ferroptosis
BP, MF, CC		HIF-1 signaling pathway
BP	Decreased	Thyroid hormone synthesis
MF	Decreased	Drug-metabolizing enzymes

## Data Availability

The original contributions presented in the study are included in the article, further inquiries can be directed to the corresponding author.
